# Multi-species suppression of herbivores through consumptive and non-consumptive effects

**DOI:** 10.1371/journal.pone.0197230

**Published:** 2018-05-23

**Authors:** Kathryn S. Ingerslew, Deborah L. Finke

**Affiliations:** Division of Plant Sciences, University of Missouri, Columbia, Missouri, United States of America; Indian Institute of Science, INDIA

## Abstract

Most studies investigating the importance of non-consumptive interactions for herbivore suppression focus on pairwise interactions between one predator and one prey, ignoring any community context. Further, the potential for non-consumptive interactions to arise between herbivores and non-enemy organisms is commonly overlooked. We investigated the relative contributions of consumptive and non-consumptive effects to aphid suppression by a wasp assemblage containing both enemies and non-enemies. We examined the suppression of two aphid species with different defensive strategies, pea aphids (*Acyrthosiphon pisum*), which drop from their host plant to the ground, and green peach aphids (*Myzus persicae*), which remain on the plant and merely walk away. The expectation was that riskier defensive behaviors, like abandoning the plant, would result in larger non-consumptive effects. We found that the outcome of multi-species interactions differed depending on the mechanism of suppression, with interference among wasps in their consumptive effects and additivity in their non-consumptive effects. We also found that, despite differences in defensive strategies, the non-consumptive effects of wasps on aphid abundance were significant for both aphid species. Furthermore, when part of a multi-species assemblage, non-enemies enhanced aphid suppression via complementary non-consumptive effects with lethal enemies, but this increase in suppression was offset by disruption in the consumptive suppression of aphids by lethal enemies. We conclude that non-consumptive effects arise from interactions with both enemy and non-enemy species and that both can contribute to herbivore suppression when part of a broader community. We predict that encouraging the presence of non-enemy organisms may provide insurance against fluctuations in the size of consumptive enemy populations and buffer against herbivore outbreaks.

## Introduction

Natural enemies suppress herbivore populations through a combination of consumptive and non-consumptive effects [1]. Consumptive effects occur when enemies kill and consume prey; non-consumptive effects arise when herbivores invest in defensive traits (e.g., behavior, morphology or physiology) in response to a perceived risk of attack [2, 3]. Although historically overlooked, non-consumptive effects often have impacts on prey populations that are equal or greater in strength to consumptive effects [1] and thus have the potential to influence the dynamics of ecological communities [4–6].

Most of our understanding of the ecological and evolutionary importance of non-consumptive effects comes from studies of pairwise species interactions [2, 7]. However, herbivores in natural communities typically interact with a diverse assemblage of predators, and the combined effect of multiple predator species on herbivore populations is rarely equal to the sum of their individual impacts [8, 9]. The few studies that have incorporated more than one predator find that the non-consumptive effects of one predator can alter the consumptive impact of other predators. For example, herbivores that respond to the presence of a foraging predator by escaping to a refuge habitat may simultaneously reduce their vulnerability to other predators in the same area (predator interference) [10] or increase their vulnerability to predators located in the refuge habitat (predator facilitation) [11, 12]. The non-consumptive effects of one predator may also alter the non-consumptive effects of other predators. Tadpoles and predatory salamanders exhibited inducible defensive/offensive phenotypes and the expression of these phenotypes was reduced by cues associated with presence of a top predator [13]. Alternatively, *Plutella xylostella* (L.) caterpillars increased the strength of their defensive behavioral response, eating less and abandoning their host plant more often, when multiple predator species were present [14]. In this case, the synergism in the non-consumptive effects of multiple predator species on herbivores cascaded down to indirectly enhance plant biomass [14]. Although there are a few notable exceptions (e.g., [10, 13, 14]), studies rarely isolate the consumptive and non-consumptive effects of predators in such a way that the contributions of each to multi-predator interactions can be rigorously investigated.

Even less appreciated is that community members that are not lethal predators can also contribute to non-consumptive effects by causing defensive changes in herbivore behavior [15]. The non-lethal organism may be a specialized natural enemy species that does not consume the particular herbivore [16], another herbivore foraging in the same environment [17], or even a pollinator. For example, caterpillars respond to the airborne vibrations produced by flying honeybees in the same way they would the vibrations of predatory wasps; they stop feeding, regurgitate, and drop from the host plant [18]. Because many insect herbivores use relatively general and indirect cues to assess potential threats (e.g., [19, 20]), the occurrence of non-consumptive effects by non-enemy organisms is potentially widespread. More studies are needed to determine how the non-consumptive effects of non-enemy organisms may interact with the consumptive and non-consumptive effects of natural enemies to influence herbivore suppression.

Our goal was to determine whether a community of arthropods that includes both enemies (i.e., consumers) and non-enemies (i.e., non-lethal organisms) results in levels of herbivore suppression greater than that accomplished by individual consumers alone. We also examined whether the magnitude of consumptive and non-consumptive suppression was different for herbivore species that differ in their defensive investment. Prey exhibit a wide variety of defensive behaviors when predation risk is perceived [2, 21, 22], and even closely-related prey can show considerable variation in their sensitivity to the presence of threats and the likelihood of engaging in risky defensive behaviors (e.g., [23]). The magnitude of the non-consumptive effects will likely be a function of the degree to which prey traits are altered in response to levels of risk [24–26]. Current modeling efforts predict that large non-consumptive effects of predators occur when prey invest heavily in defense [27].

Pea aphids (*Acyrthosiphon pisum* (Harris)) (Hemiptera: Aphididae) possess a dramatic escape behavior of dropping from their host plant when threatened [28–30]. This defensive behavior enhances the likelihood that pea aphids survive an enemy encounter [31], but can indirectly reduce fitness due to the reduction in time spent feeding and increased chance of mortality from abiotic and biotic conditions on the ground [11, 32, 33]. This tradeoff is evolutionarily advantageous when faced with natural enemies like the parasitoid wasp *Aphidius ervi* Haliday (Hymenoptera: Braconidae), an efficient consumer of pea aphids [34]. However, pea aphids also exhibit this escape behavior in response to the presence of *Aphidius colemani* Viereck, a species of parasitoid wasp that does not consume pea aphids [16, 35]. Pea aphids suffer additional non-consumptive declines in fitness due to handling by both wasp species as they assess aphid suitability as prey, resulting in injury when attacks are unsuccessful [36]. While pea aphids are only vulnerable to consumption by *A*. *ervi*, the existence of strong non-consumptive effects means that both *A*. *ervi* and the non-lethal wasp, *A*. *colemani*, cause significant reductions in pea aphid population size when their independent effects are assessed using pairwise interaction studies [16, 36].

Alternatively, green peach aphids (*Myzus persicae* (Sulzer)) (Hemiptera: Aphididae) are consumed by both wasp species and they exhibit relatively subtle behavioral responses to the threat of parasitism [35, 37]. Green peach aphids rarely drop from their host plant when threatened and tolerate the physical trauma of non-deadly interactions with wasps (e.g., failed attacks) without significant declines in performance [36]. They do occasionally interrupt their feeding to walk away from wasps, but this is not a consistent behavioral defense. Therefore, unlike pea aphids, green peach aphids are vulnerable to the consumptive and non-consumptive effects of both wasp species, but given their relatively moderate behavioral responses, any suppression by parasitoid wasps in pairwise interaction studies is largely the result of consumptive effects [36].

**Fig 1:**
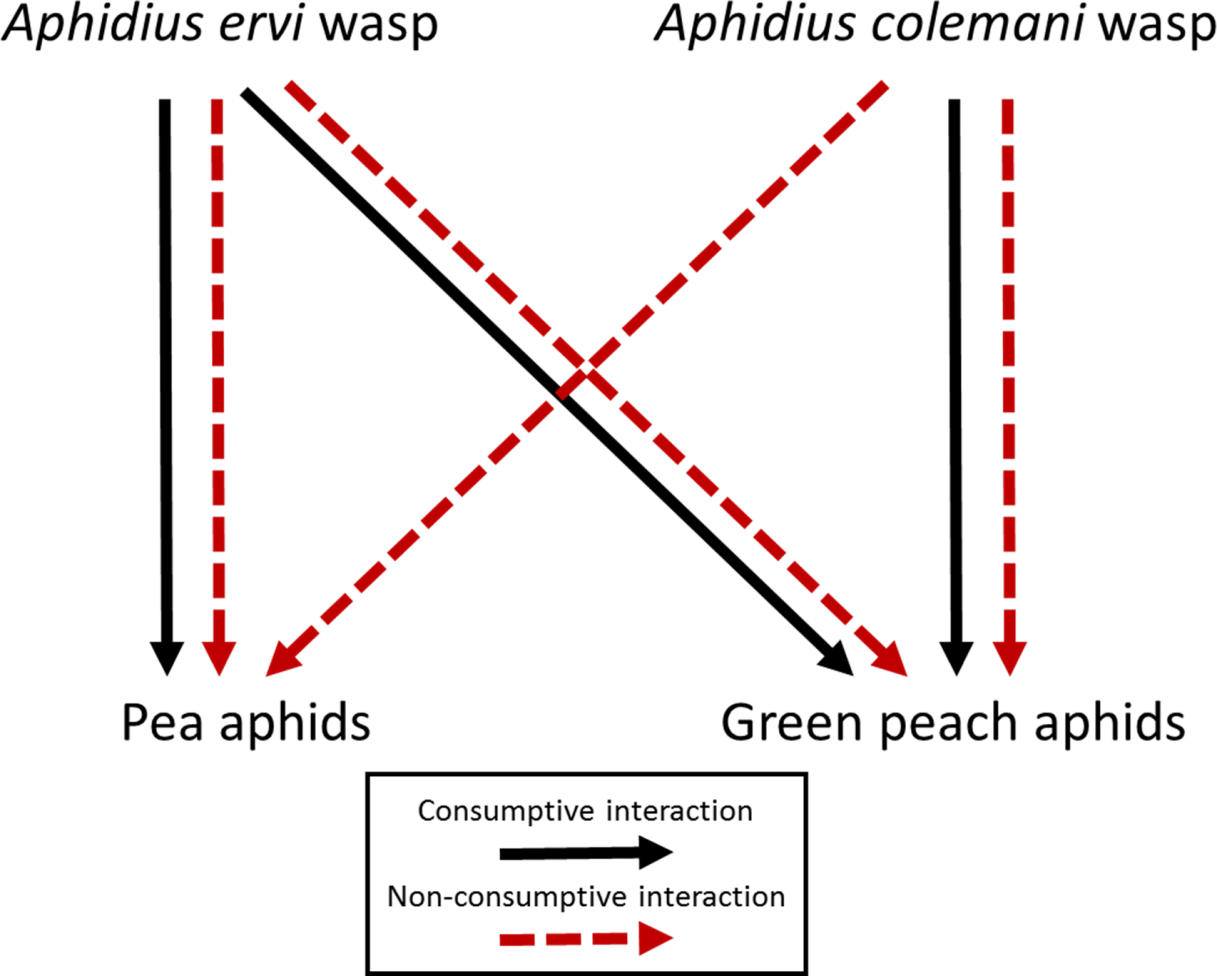
Interaction web showing direct interactions between the parasitoid wasps *Aphidius ervi* and *Aphidius colemani* and the aphid hosts *Acyrthosiphon pisum* (pea aphid) and *Myzus persicae* (green peach aphid). Black, solid arrows indicate consumptive interactions (i.e., aphid population declines due to parasitism). Red, dashed arrows indicate non-consumptive interactions (i.e., population declines due to aphid defensive investment). Note that *A*. *colemani* does not parasitize pea aphids, but still influences pea aphid abundance through non-consumptive interactions.

In this study, we investigated the contributions of consumptive and non-consumptive effects to aphid suppression by two parasitoid wasp species simultaneously (Fig 1). We asked: (1) how do the consumptive and non-consumptive effects of the two wasps combine to influence pea aphid and green peach aphid suppression? We were specifically interested in whether the non-consumptive suppression of pea aphids by the non-enemy species, *A*. *colemani*, is complementary to the suppression of pea aphid populations by the enemy, *A*. *ervi*. Our hypothesis was that the wasps *A*. *ervi* and *A*. *colemani* will additively suppress pea aphid and green peach aphid populations through a combination of consumptive and non-consumptive effects. We also asked: (2) does the relative importance of consumptive and non-consumptive suppression by the two wasp species differ for aphids with different investments in defense? We hypothesized that non-consumptive effects will play a relatively larger role in suppression of pea aphids than green peach aphids, given that pea aphids have a stronger behavioral response to the presence of perceived threats.

Placing non-enemy interactions within a community framework will contribute to our understanding of the dynamics of ecological communities and provide critical information on the usefulness and generality of non-consumptive suppression for managing herbivore pests in agroecosystems. We have shown previously that non-consumptive suppression of herbivores by non-enemies is possible when this interaction is examined in isolation [16, 36]. Whether we can take advantage of non-enemies to significantly enhance herbivore suppression in the field will ultimately depend on the relative importance of these interactions when placed within the context of a community including consumers.

## Materials/Methods

### Experiment 1 –Additive design

This study was conducted to determine how the wasps *A*. *ervi* and *A*. *colemani* interact to suppress pea and green peach aphid populations. The study was conducted at the University of Missouri Ashland Road Greenhouse facility (Columbia, MO, 16:8 L:D, 26–38°C). Experimental units were 60 cm x 60 cm x 60 cm bug dorm insect cages (MegaView Science Co., Ltd., Taichung, Taiwan). Inside each cage we placed a 27 cm x 54 cm planting tray containing one row of five 10-day-old fava bean plants (*Vicia faba* L.) and one row of five 19-day-old collard plants (*Brassica oleracea* L.) that were spaced approximately 7 cm apart. Aphids were added to their preferred host plant: 50 pea aphids were haphazardly added to the row of fava bean plants and 50 green peach aphids were haphazardly added to the row of collard plants, for an average of 10 aphids per plant. Aphids remained separated on their preferred host plants throughout the study (S1 Fig). After a 24-h settling period for the aphids, one of four wasp treatments was randomly assigned: (1) control with no wasps present, (2) 25 adult *A*. *ervi*, (3) 25 adult *A*. *colemani*, or (4) a mixture of 25 adult *A*. *ervi* and 25 adult *A*. *colemani* (Rincon-Vitova Insectaries, Ventura, California, US, sex ratio 1:1). Treatments were replicated seven times. This additive manipulation of wasp presence doubled the overall abundance of wasps when both species were present.

After 7 d, we counted the number of pea aphids and green peach aphids present. After 14 d, we counted the number of pea aphid and green peach aphid mummies present. Fourteen days is ample time to allow for formation of both *A*. *ervi* and *A*. *colemani* mummies [38, 39]. All mummies were collected for wasp species identification after emergence.

#### Estimating consumptive and non-consumptive effects

Consumptive effects are measured as the number of prey killed and consumed by an enemy, thus it can be difficult to quantify the consumptive effects for each individual species in an enemy assemblage without direct observation of who eats whom. Parasitoid wasps do not have the same difficulty in quantifying consumptive effects because they have a unique strategy to kill and consume their prey. Female adult wasps use their ovipositor to insert an egg inside the aphid body. The egg hatches and the wasp larva feeds within the living aphid until the wasp eventually pupates and the aphid dies. At pupation the aphid body forms a distinctive ‘mummy’ or a case surrounding the cocoon of the wasp pupa [40, 41]. The identity of the adult that emerges from the pupa reveals the identity of the consumer. Therefore, we quantified the consumptive effect of the wasps, the number of aphids consumed, by counting the number of mummies that formed. The non-consumptive effect of the wasps, population decline not due directly to parasitism, was quantified as any decline in aphid population size that was not accounted for by mummy formation. To estimate this, we compared aphid population size in the presence and absence of wasps after ample time for interactions and parasitism to occur, but prior to mummy formation and aphid death (7 days). Therefore, an increase in the magnitude of the non-consumptive effect of wasps was reflected in a decline in aphid abundance at day 7, while an increase in the magnitude of the consumptive effect was reflected in an increase in the number of mummies that form.

#### Statistical analyses

The main and interactive effects of *A*. *ervi* and *A*. *colemani* on the reduction in pea aphid and green peach aphid abundance due to non-consumptive effects (i.e., aphid abundance on day 7) were analyzed in two separate two-way analyses of variance (ANOVA) (PROC MIXED, SAS v.9.3, SAS Institute, Cary, NC) using log-transformed aphid abundance. A multiplicative risk model was used to avoid overestimation of the expected aphid population reduction in treatments where both wasps were present [12, 42]. The consumptive effect (i.e., the number of mummies that formed) was compared with one-way ANOVAs, using ‘wasp treatment’ as the predictive variable. Pea aphids are only parasitized by *A*. *ervi*, therefore ‘wasp treatment’ for the pea aphid analysis compared mummy abundance when only *A*. *ervi* was present to the treatment when both *A*. *ervi* and *A*. *colemani* were present. Green peach aphids are parasitized by both wasps, so ‘wasp treatment’ compared mummy abundance across the three treatments where wasps were present (*A*. *colemani* alone, *A*. *ervi* alone, and a mix of *A*. *colemani* and *A*. *ervi*). Since *A*. *ervi* parasitize both aphid species, we also compared the distribution of *A*. *ervi* reproductive effort across aphids (i.e., the proportion of adult *A*. *ervi* that emerged from pea aphid mummies versus green peach aphid mummies) in the presence and absence of *A*. *colemani*. A cluster of four cages experienced high aphid mortality due to abiotic environmental conditions in the greenhouse and were not included in any analyses.

### Experiment 2 –Substitutive design

We manipulated the presence of *A*. *ervi* and *A*. *colemani* in a substitutive/replacement design using an identical experimental set-up to Experiment 1, but in this case, we maintained a constant total density of wasps across all treatments by reducing the abundance of each species when present together. Wasp treatments were as follows: (1) control with no wasps present, (2) 20 *A*. *ervi*, (3) 20 *A*. *colemani*, or (4) a mixture of 10 *A*. *ervi* and 10 *A*. *colemani* (Rincon-Vitova Insectaries, Ventura, California, US, sex ratio 1:1). Each treatment was replicated six times.

The consumptive and non-consumptive effects of wasps were determined as described above. In addition, the *per capita* mummy production of each wasp species (number of mummies/number of wasps) was determined to facilitate a direct comparison of the consumptive effects of each individual wasp species when present alone (20 conspecifics) versus when present in the mixture with the other wasp species (10 conspecifics).

#### Statistical analyses

The non-consumptive effects of *A*. *ervi* and *A*. *colemani* on the pea aphid and green peach aphid populations were compared across the four wasp treatments in two separate one-way ANOVAs using ‘treatment’ as the predictive variable. To determine whether the effects of the wasps in combination were different than expected based on the strength of the effects when each wasp was present alone, an orthogonal contrast was used to compare the pooled effect of *A*. *ervi* and *A*. *colemani* treatments to that of the mixed treatment (CONTRAST, PROC MIXED, SAS v.9.3, SAS Institute, Cary, NC). The consumptive effects of *A*. *ervi* and *A*. *colemani* (*per capita* mummy formation) were compared when each was alone versus present with the other wasp using two one-way ANOVAs, with the two aphid species analyzed separately. We also analyzed total green peach aphid mummy formation across treatments where wasps were present using a one-way ANOVA. Since *A*. *ervi* parasitizes both aphid species, we also compared the distribution of *A*. *ervi* reproductive effort across aphids (i.e., the proportion of adult *A*. *ervi* that emerged from pea aphid mummies versus green peach aphid mummies) in the presence and absence of *A*. *colemani*. To meet the assumptions of ANOVA, the non-consumptive effect of wasps on the green peach aphid population was log-transformed.

## Results

### Experiment 1 –Additive design

#### Pea aphid

**Fig 2:**
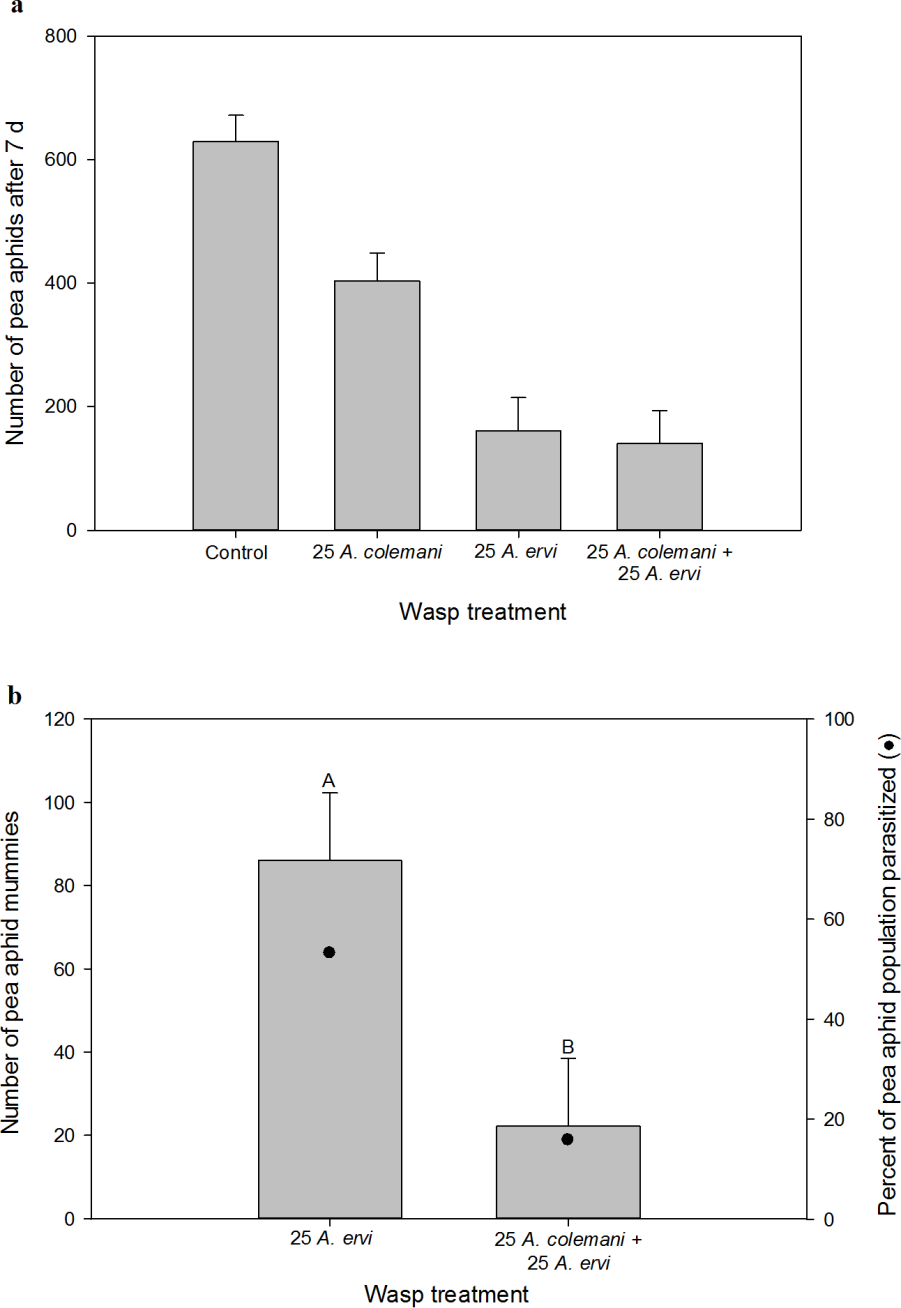
Experiment 1, *pea aphids*. (a) The non-consumptive effect of parasitoid treatment on pea aphid abundance seven days after parasitoid introduction. LS means + 1 SEM for all treatments are shown, but no comparisons of means were performed because the interaction was not significant (main effect of *A*. *ervi p* < 0.05, main effect of *A*. *colemani p* < 0.10). (b) The consumptive effect of parasitoid treatment on pea aphid abundance, measured by the production of mummies (grey bars) and percent parasitism of the pea aphid population (●). *Aphidius ervi* parasitizes pea aphids and *A*. *colemani* does not. Only treatments where pea aphid mummies formed are shown. LS means + 1 SEM are shown. Letters indicate significant differences at the α = 0.05 level.

Pea aphids, which respond to the risk of predation by dropping from their host plant, experienced significant non-consumptive suppression by the lethal enemy *A*. *ervi* (*F*_1,21_ = 35.53, *P* < 0.0001, Fig 2A) and moderately significant non-consumptive suppression by the non-enemy *A*. *colemani* (*F*_1,21_ = 3.06, *P* = 0.09, Fig 2A). There was no evidence of an interaction between the wasps in their non-consumptive effects on the pea aphid population (*F*_1,21_ = 0.12, *P* = 0.73, Fig 2A).

The consumptive effect of the pea aphid enemy *A*. *ervi*, as measured by the formation of mummies, was negatively influenced by the presence of the non-enemy *A*. *colemani* (*t*_8_ = 7.69, *P* = 0.024, Fig 2B). The presence of *A*. *colemani* caused a >75% decline in parasitism of pea aphids by *A*. *ervi*. This was due to an overall decline in parasitism by *A*. *ervi* in the presence of *A*. *colemani*, and not due to a shift in the allocation of *A*. *ervi* reproductive effort between pea aphids and green peach aphids (*t*_7_ = 0.52, *P* = 0.6188, S2A Fig). The rate of pea aphid parasitism was moderate to low in both treatments, with *A*. *ervi* parasitizing 53% of the population when alone 16% of the population when present with the non-enemy. Identification of adult wasps after emergence confirmed that 100% of pea aphid mummies were *A*. *ervi*.

#### Green peach aphid

**Fig 3:**
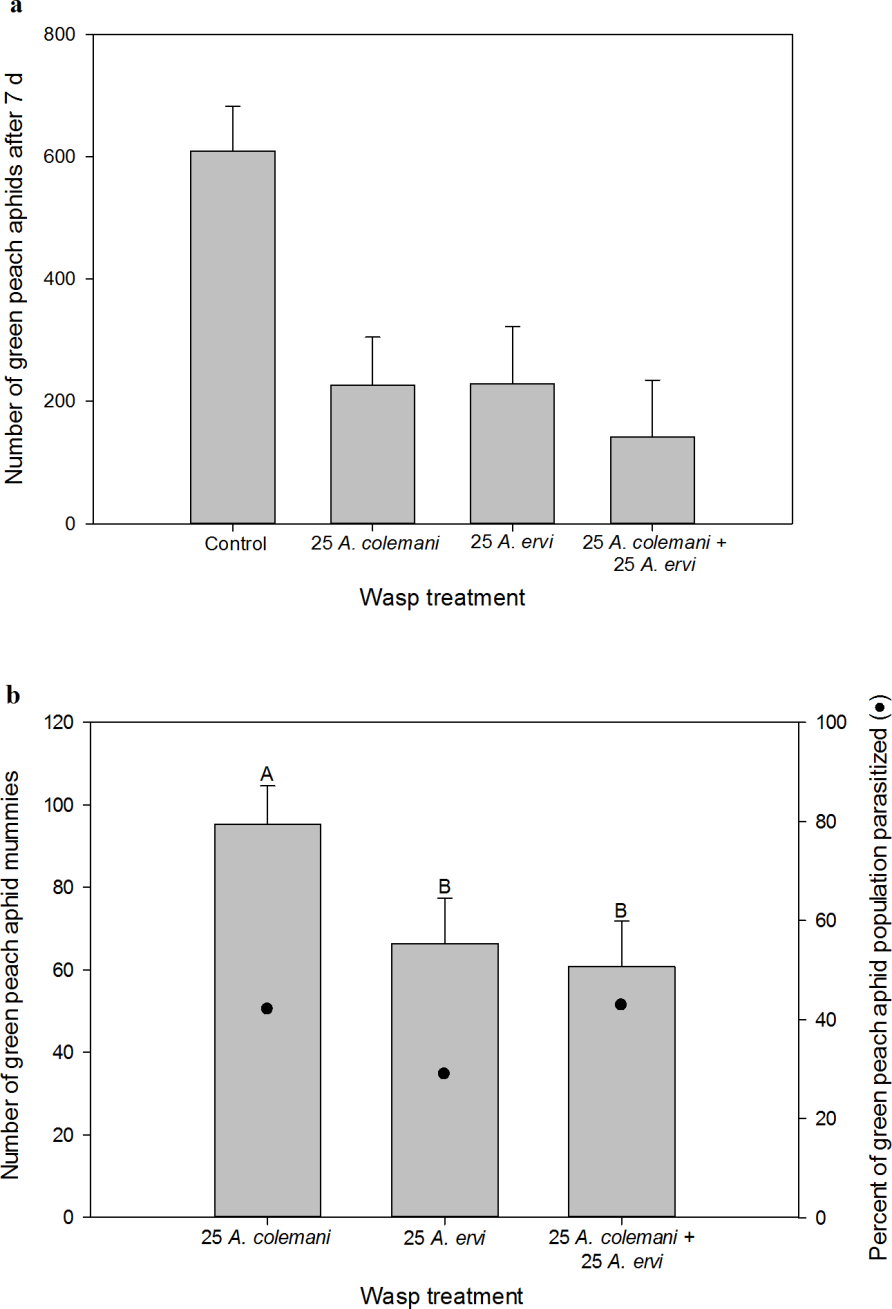
Experiment 1, *green peach aphids*. (a) The non-consumptive effect of parasitoid treatment on green peach aphid abundance seven days after parasitoid introduction. The main effects of both *A*. *ervi* and *A*. *colemani* were significant at α = 0.05. (b) The consumptive effect of parasitoid treatment on green peach aphid abundance, measured by the production of mummies. *Aphidius ervi* and *A*. *colemani* both parasitize green peach aphids. Only treatments where green peach aphid mummies formed are shown. LS means + 1 SEM are shown. Letters indicate significant differences at the α = 0.05 level.

Green peach aphids, despite only moderate behavioral responses to the threat of predation, declined in abundance as a result of non-consumptive interactions with the wasps *A*. *ervi* and *A*. *colemani* (*t*_21_ = 3.51, *P* = 0.0021, *t*_21_ = 4.12, *P* = 0.0005, respectively; Fig 3A). The magnitude of non-consumptive suppression by each of the wasps was equivalent and not affected by the presence of the other wasp (*F*_1,21_ = 1.89, *P* = 0.184).

Both wasp species are lethal enemies of green peach aphids, but the magnitude of their consumptive effects differed (*F*_2,14_ = 3.49, *P* = 0.059; Fig 3B). When alone, parasitism of green peach aphids by *A*. *colemani* was slightly greater than that by *A*. *ervi* (*t*_14_ = 2.01, *P* = 0.064). When present together in the mixed treatment, there was evidence of antagonism between the wasp species. Mummy abundance as a result of parasitism by both wasps was lower than when *A*. *colemani* was present alone (*t*_14_ = 2.40, *P* = 0.031) and the same as when *A*. *ervi* was present alone (*t*_14_ = 0.36, *P* = 0.724), despite having twice as many wasps present. The majority of green peach aphid parasitism in the mixed treatment was attributed to *A*. *colemani*, with adult *A*. *colemani* emerging from 84% of the mummies. Only 16% of the adults emerging from green peach aphid mummies collected from the mixed treatment were *A*. *ervi*. Again, the reduction in green peach aphid parasitism by *A*. *ervi* in the presence of *A*. *colemani* did not reflect a shift to parasitism of pea aphids, but an overall reduction in parasitism by *A*. *ervi*, since the allocation of *A*. *ervi* reproductive effort between green peach aphids and pea aphids was not different (S2A Fig). Parasitism rates of green peach aphids were moderate across all treatments, as 42, 29, and 43% of the green peach aphid population was parasitized when *A*. *colemani* was alone, *A*. *ervi* was alone, and in the mixed treatment, respectively.

### Experiment 2 –Substitutive design

#### Pea aphid

**Fig 4:**
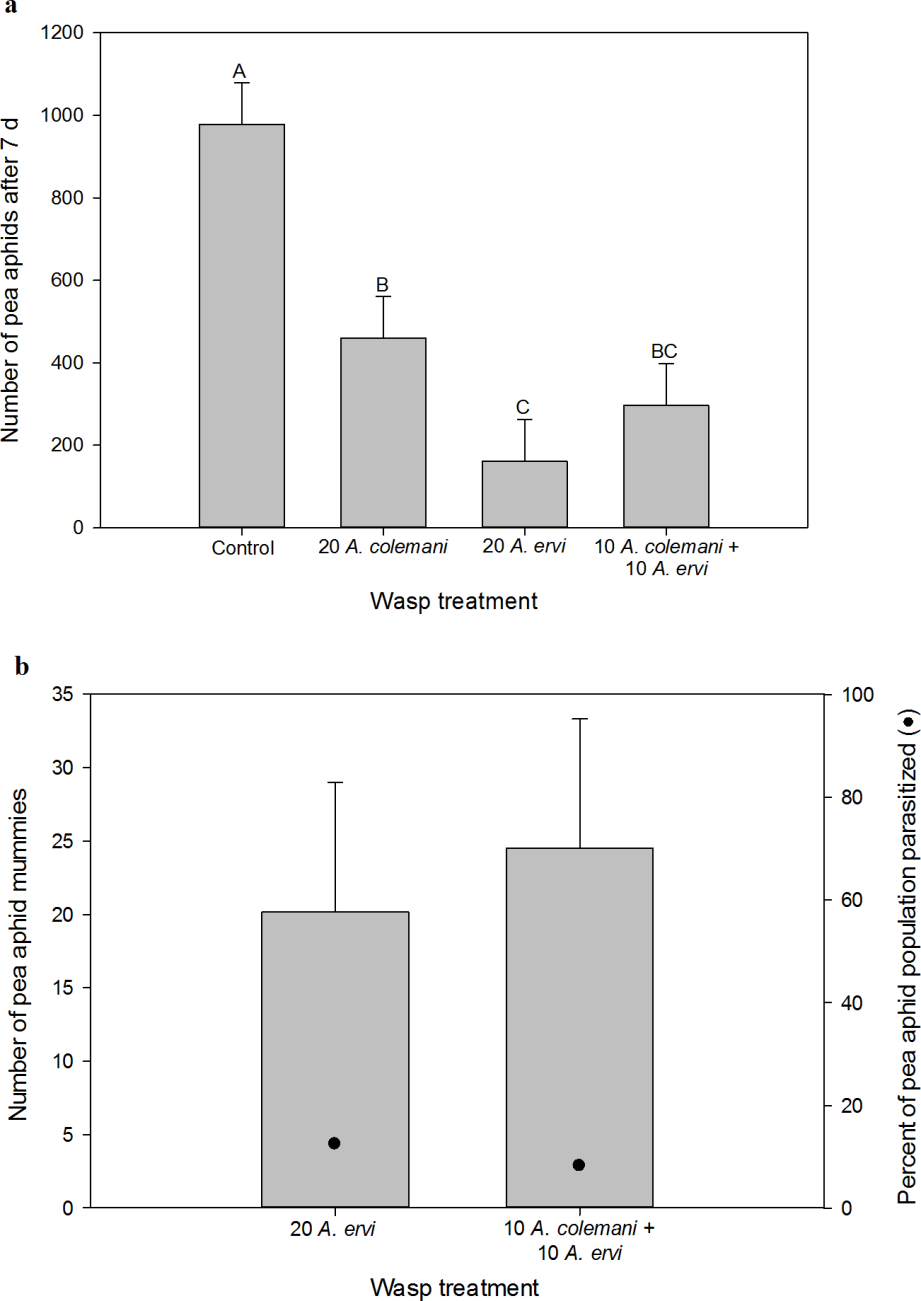
Experiment 2, *pea aphids*. (a) The non-consumptive effect of parasitoid treatment on pea aphid abundance seven days after parasitoid introduction. (b) The consumptive effect of parasitoid treatment on pea aphid abundance, measured by the production of mummies. *Aphidius ervi* parasitizes pea aphids and *A*. *colemani* does not. Only treatments where pea aphid mummies formed are shown. LS means + 1 SEM are shown. Letters indicate significant differences at the α = 0.05 level.

The non-consumptive effects of the wasps on the pea aphid populations varied across the four treatments (one-way ANOVA: *F*_3,20_ = 12.53, *P* < 0.001, Fig 4A). Both wasps suppressed pea aphid abundance, but the lethal enemy *A*. *ervi* had a larger non-consumptive effect than the non-enemy *A*. *colemani* (*t*_20_ = 2.09, *P* = 0.050). When both wasps were present together and total density was held constant, the non-consumptive effects of the wasps on pea aphids were additive. The pooled average of the effects of each wasp alone was not different from the effects of the mixed treatment where both were present together (*t*_20_ = 0.01, *P* = 0.914).

The consumptive effect of *A*. *ervi* on the pea aphid population was not affected by the presence of the non-enemy *A*. *colemani* when the experimental design held wasp density constant. Pea aphid mummy formation was not different in the two treatments where enemy wasps were present (*t*_10_ = 0.35, *P* = 0.7353, Fig 4B). Additionally, we saw no difference in *per capita* mummy production by *A*. *ervi* when alone as compared to when present with *A*. *colemani* (1.01 ± 0.73 versus 2.38 ± 0.73 mummies per adult *A*. *ervi*, respectively; *t*_10_ = 1.33, *P* = 0.214). Furthermore, the presence of *A*. *colemani* did not change the allocation of *A*. *ervi* parasitism between pea aphids and green peach aphids (*t*_10_ = 1.57, *P* = 0.1482, S2B Fig). Overall parasitism rates were low, with *A*. *ervi* parasitizing 12% of the population when alone and 8% of the population when with the non-enemy *A*. *colemani*.

#### Green peach aphid

**Fig 5:**
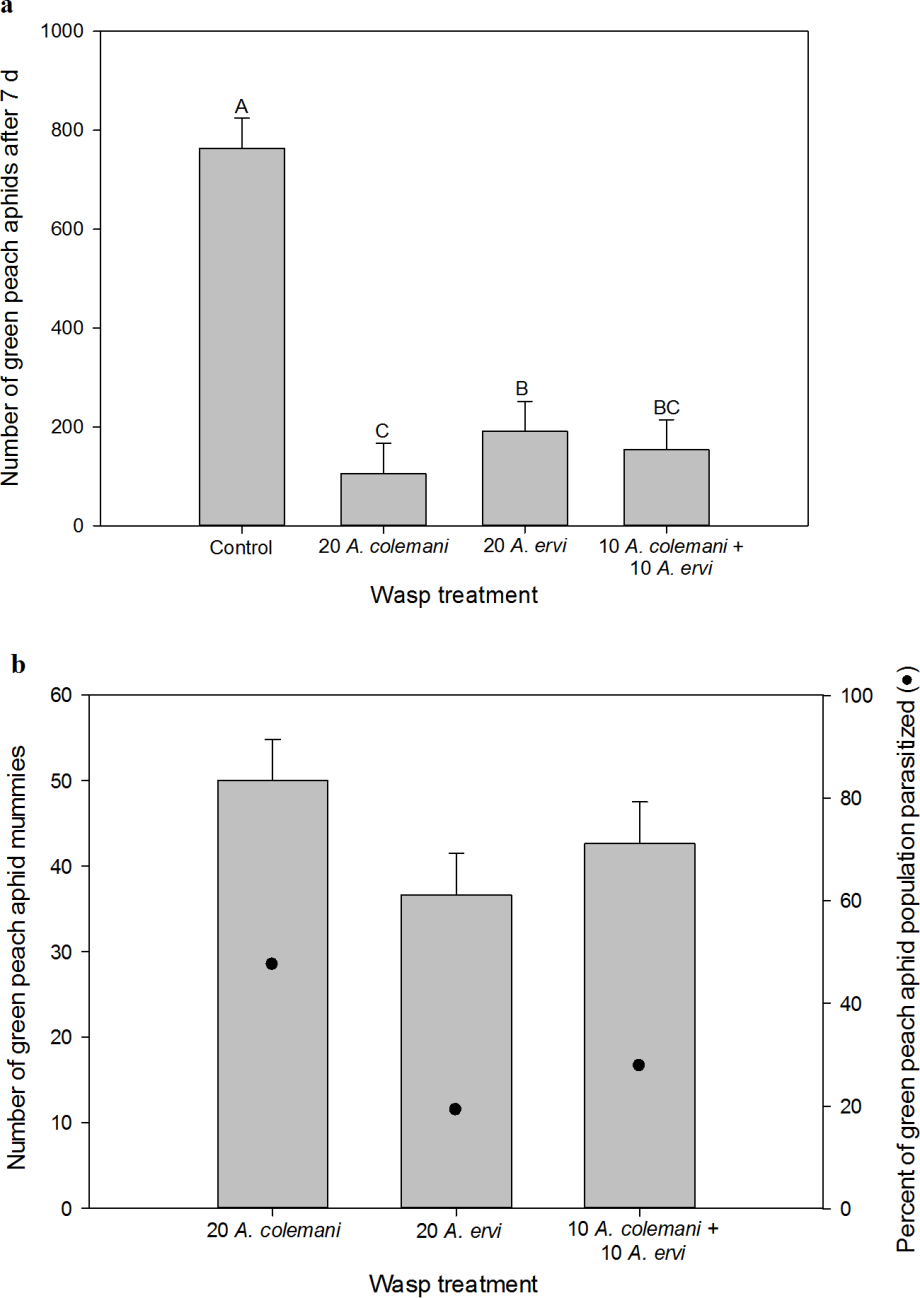
Experiment 2, *green peach aphids*. (a) The non-consumptive effect of parasitoid treatment on green peach aphid abundance seven days after parasitoid introduction. (b) The consumptive effect of parasitoid treatment on green peach aphid abundance, measured by the production of mummies. *Aphidius ervi* and *A*. *colemani* both parasitize green peach aphids. Only treatments where green peach aphid mummies formed are shown. LS means + 1 SEM are shown. Letters indicate significant differences at the α = 0.05 level.

The non-consumptive effects of wasps on the green peach aphid populations varied across treatments (one-way ANOVA: *F*_3,20_ = 19.59, *P* < 0.001, Fig 5A). Both *A*. *colemani* and *A*. *ervi* suppressed green peach aphids compared to the control (*t*_20_ = 7.29, *P* < 0.001 and *t*_20_ = 4.99, *P* < 0.001, respectively), but *A*. *colemani* suppressed green peach aphids to a greater degree than *A*. *ervi* (*t*_20_ = 2.30, *P* = 0.033). Furthermore, the pooled average of the effects of each wasp species alone was not different from the mixed treatment where both species were present together (*F*_1,20_ = 0.46, *P* = 0.504), indicating additivity in the non-consumptive effects of the two wasps on green peach aphid abundance.

Total green peach aphid mummy formation (consumptive effect) was not different across treatments containing wasps (*F*_2, 15_ = 1.89, *P* = 0.186, Fig 5B). Additionally, we saw no difference in *per capita* mummy production by *A*. *colemani* when alone as compared to when present with *A*. *ervi* (2.50 ± 0.62 versus 2.93 ± 0.62 mummies per adult *A*. *colemani*, respectively; *t*_10_ = 0.49, *P* = 0.633) or *per capita* mummy production by *A*. *ervi* when alone as compared to when present with *A*. *colemani* (1.83 ± 0.27 versus 1.34 ± 0.27 mummies per adult *A*. *ervi*, respectively; *t*_10_ = 1.32, *P* = 0.217). Parasitism rates of green peach aphids were moderate across all treatments, as 48, 19 and 28% of the green peach aphid population was parasitized when *A*. *colemani* was alone, *A*. *ervi* was alone, and in the mixed treatment, respectively.

## Discussion

Most studies of non-consumptive predator effects on herbivores limit their investigations to pairwise species interactions [2, 7]. However, herbivores in natural communities typically interact with a diverse assemblage of both enemy and non-enemy organisms that can all impact herbivores through non-consumptive effects [8, 9, 16–18]. We examined how the consumptive and non-consumptive effects of two wasp species combine to influence the suppression of two aphid species that differ in their defensive strategies, with specific interest in determining whether non-enemies can contribute to the management of herbivorous pests in agroecosystems.

From a consumptive perspective, we found that conclusions about the outcome of the multi-species interaction differed depending on the nature of the wasp treatment manipulation. An additive design, where the presence of interspecific interactions was confounded with an increase in overall wasp abundance, revealed antagonism between the wasps. For pea aphids and green peach aphids, the number of mummies that formed when both wasps were present (and total wasp abundance was doubled) was less than predicted based the effects of each wasp alone. However, when total wasp abundance was held constant across treatments, the effects of the two wasps were found to be substitutable. In this case, the strength of intraspecific and interspecific interactions among wasps were equivalent [43]. Together, these results suggest that interference occurs among wasps in their consumptive effects on aphids, but the relative impact of interspecific and intraspecific interference is the same [44]. Therefore, the observed reduction in consumptive effects when using the additive treatment design was the result of an increase in the intensity of interference due to an increase in overall wasp density, rather than the addition of another species *per se* [44]. This interference was likely behavioral, with wasps avoiding visual or chemical cues associated with con- or heterospecific wasps [45]. Interference may also arise from resource over-exploitation, resulting in redundant attacks on aphids through super- or multi-parasitism [46–49]. However, this type of interference was unlikely given that parasitism rates were low to moderate in all cases. No matter what the mechanism, we infer that our caged experimental arenas likely intensified antagonistic interactions among wasps and that interference among individuals may be less common in nature where communities are not spatially constrained [50].

We found no evidence of antagonism among wasps from a non-consumptive perspective. The magnitude of aphid suppression scaled with density in a multiplicative fashion, and the intensity of inter- and intraspecific interactions were equal such that the effects of the two wasp species were substitutable. While it appears that behavioral interference reduced parasitism of aphids by wasps, it does not appear to have affected the ability of wasps to stimulate the defensive responses of aphids. At least one other study has found that the non-consumptive effects of multiple enemy species combine to enhance herbivore suppression, while multi-enemy consumptive effects do not [14], and this can be true for the indirect effects of enemies on plants as well [51]. Differences in how the consumptive and non-consumptive effects of natural enemies combine to affect herbivore suppression may be due to the fact that enemies can kill only one herbivore at a time, but can simultaneously ‘scare’ numerous individuals [14, 52]. This is especially true for aphids, which produce an alarm pheromone to warn conspecifics of the presence of a threat [53]. Therefore, while restricted foraging to avoid con- or hetero-specific wasps may limit the number of aphids that are susceptible to consumption via parasitism, non-consumptive effects may still permeate beyond the boundaries of the wasp foraging area because aphids have the ability to detect and respond to threats from a distance [54].

We predicted that non-consumptive effects would be relatively more important for herbivores like pea aphids, which are easily disturbed and frequently engage in dramatic (and costly) enemy-avoidance behaviors. However, we also found large non-consumptive effects of wasps on green peach aphids, which merely walk away from the threat of predation [36]. Although walking away appears to be a relatively conservative defensive strategy as compared to dropping off of the host plant, it still requires the aphid to cease feeding and find another suitable feeding site, which can be fairly time intensive as it involves navigating their mouthparts around the cell membranes of a plant to access the phloem [55]. Hence, it is not surprising that significant non-consumptive effects of parasitoid wasps on green peach aphid populations were found. The fact that non-consumptive effects are not unique to herbivores with dramatic responses to the threat of predation, and even herbivores with subtle defensive techniques still suffer fitness costs, is consistent with the finding that non-consumptive effects appear to be present in almost every predator and prey relationship studied (e.g., [6]) and are usually just as significant and maybe more significant than the consumptive effects of predators on suppression of herbivores [1].

A secondary goal of this study was to determine whether non-enemies complement herbivore suppression by enemies and enhance suppression beyond the level accomplished by consumers alone. We corroborated our previous finding that the non-enemy wasp *A*. *colemani*, which does not consume pea aphids, is capable of reducing pea aphid abundance on its own by provoking costly defensive behaviors (but not to the same magnitude as the actual enemy *A*. *ervi*) [16, 36]. Furthermore, the addition of the non-enemy to a system already containing the enemy *A*. *ervi* augmented non-consumptive suppression of pea aphids in an additive fashion, suggesting complementary suppression by the two wasps is possible. However, we also found evidence to suggest that the addition of the non-enemy wasp, *A*. *colemani*, may interfere with consumptive parasitoid-host dynamics by reducing the rate of pea aphid parasitism by *A*. *ervi*. This interference was only seen when the addition of the non-enemy was confounded with an overall increase in wasp abundance; there was no change in the *per capita* rate of pea aphid parasitism by *A*. *ervi* in the presence of the non-enemy when the total abundance of wasps did not change. These results suggest that the overall increase in wasp density, rather than the addition of the non-enemy *per se*, resulted in the lower number of pea aphid mummies and that the observed antagonism may be an artifact of the cage. Additional studies at ecologically relevant spatial and temporal scales are necessary to understand the contribution of non-enemies to herbivore suppression in natural communities [56].

It is becoming increasingly clear that pairwise species interaction studies based solely on who eats whom may not adequately describe the complex population and community dynamics contributing to herbivore suppression (e.g., [15, 57, 58]). Non-consumptive effects provide the opportunity for organisms to expand their ‘zone of influence’ within the community by directly affecting the abundance of herbivore species that they do not consume [16–18]. Further, expanding studies of non-consumptive effects beyond simple pairwise species interactions has the potential to reveal emergent outcomes and more accurately predict community dynamics (e.g., [10–14]). More recently, it has been suggested that non-consumptive interactions between predators and their prey could be exploited to enhance the natural control of herbivorous pests in agricultural systems [16, 36, 59–62] and that management efforts targeted specifically on enemies that directly consume prey may not exploit the full impact of the ecological community on pest populations [63, 64]. We have shown that a non-enemy organism has the potential to influence herbivore population size purely through behavioral, non-consumptive interactions, but to understand the contribution of non-threat organisms to herbivore suppression in natural and agricultural systems will require further study at greater spatial and temporal scales [56].

## Supporting information

S1 Fig Percentage of pea aphids (PA) and green peach aphids (GPA) found on host plants or on the cage at day 7 of the experiment. Each cage contained a row of fava bean plants and a row of collard plants. Aphids were originally released on their preferred host plant, pea aphids on fava beans and green peach aphids on collards. After 7 d, the majority of aphids were still found on their preferred host, 96.55% of pea aphids were found on fava bean plants and 99.88% of green peach aphids were found on collard host plants. In total, 9,365 pea aphids and 8,053 green peach aphids were counted.
(TIF)

S2 Fig Proportion of *Aphidius ervi* parasitism allocated to green peach aphids versus pea aphids in the presence and absence of *A*. *colemani*. Experiment 1 (a) and Experiment 2 (b). There was no difference in the allocation of *A*. *ervi* parasitism between the two aphids in the presence of *A*. *colemani* in Experiment 1 or Experiment 2 (*t*_7_ = 0.52, *P* = 0.6188; *t*_10_ = 1.57, *P* = 0.1482, respectively).
(TIF)

S1 Table Dataset. (XLSX)
