# Supplementary figures and images for: Multi-species suppression of herbivores through consumptive and non-consumptive effects
Source: PLoS One. 2018 May 23;13(5):e0197230. doi: 10.1371/journal.pone.0197230 (PMC5965886; doi:10.1371/journal.pone.0197230)

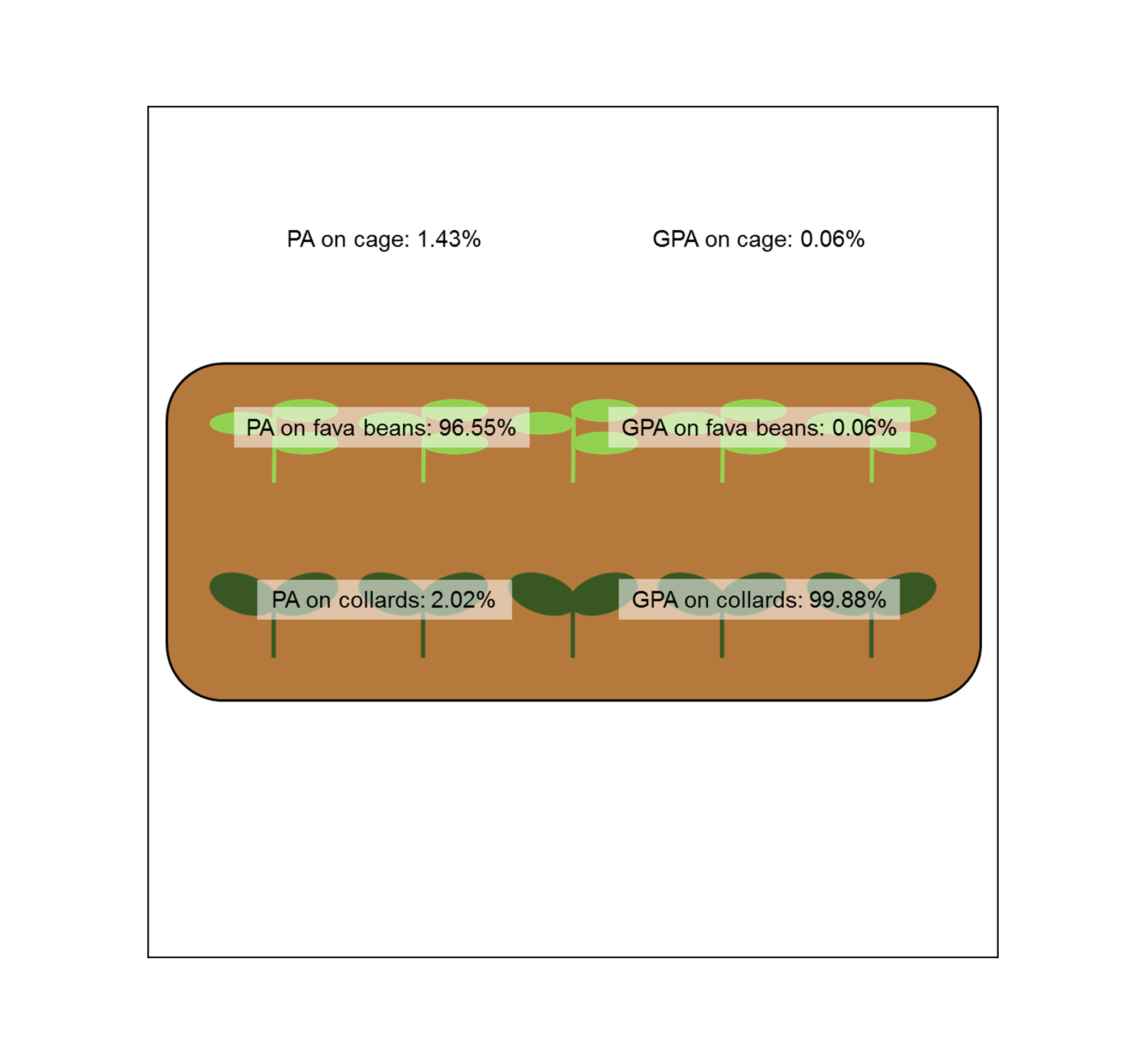

Supplement: S1 Fig — Each cage contained a row of fava bean plants and a row of collard plants. Aphids were originally released on their preferred host plant, pea aphids on fava beans and green peach aphids on collards. After 7 d, the majority of aphids were still found on their preferred host, 96.55% of pea aphids were found on fava bean plants and 99.88% of green peach aphids were found on collard host plants. In total, 9,365 pea aphids and 8,053 green peach aphids were counted. (TIF) [file pone.0197230.s001.tif]

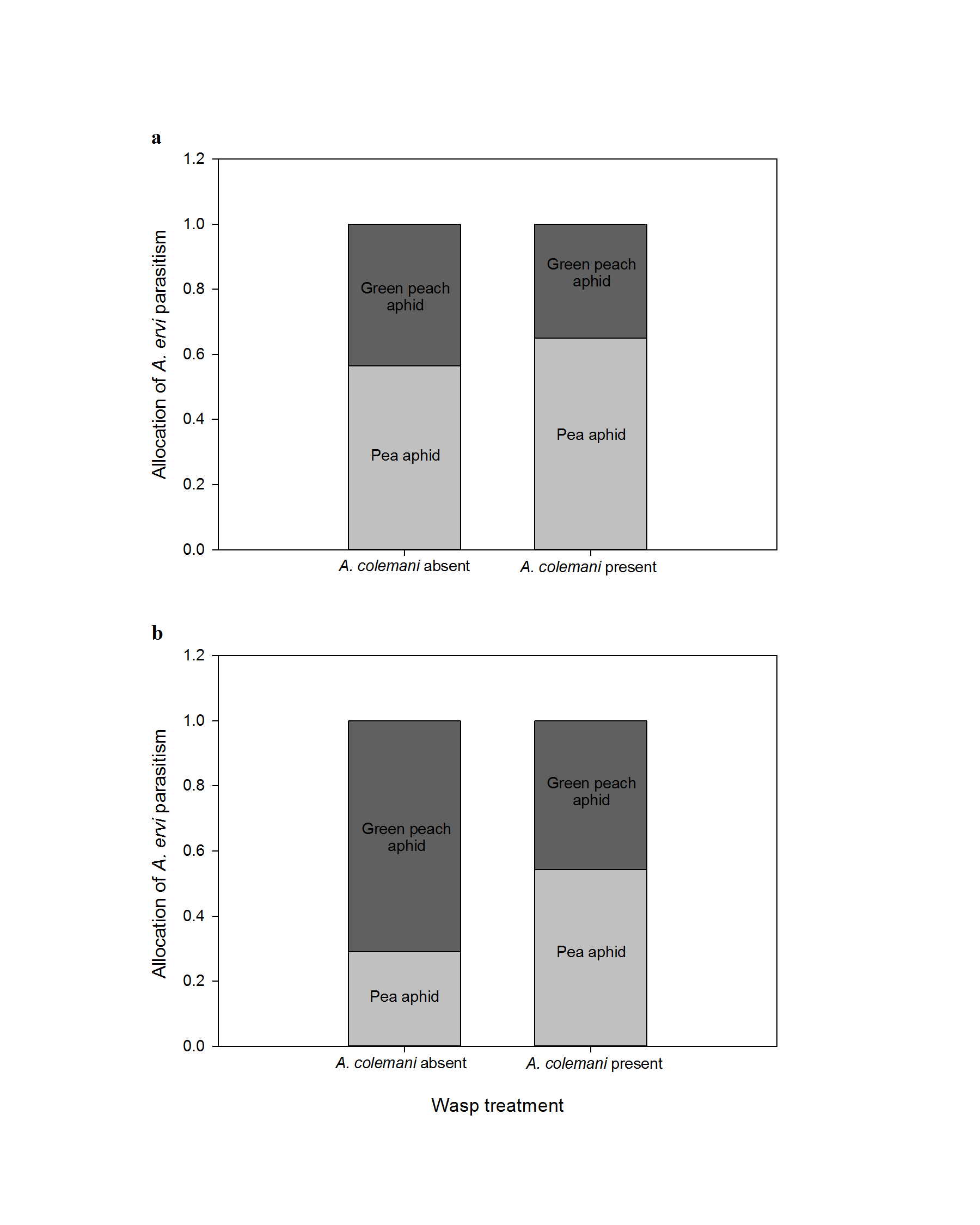

Supplement: S2 Fig — Experiment 1 (a) and Experiment 2 (b). There was no difference in the allocation of A. ervi parasitism between the two aphids in the presence of A. colemani in Experiment 1 or Experiment 2 (t7 = 0.52, P = 0.6188; t10 = 1.57, P = 0.1482, respectively). (TIF) [file pone.0197230.s002.tif]
